# Diagnostic Use of Testing for Novel Murine Autoantibodies for Sjögren Disease in the Rheumatology Outpatient Setting

**DOI:** 10.1002/acr.70005

**Published:** 2026-03-02

**Authors:** Chadwick R. Johr, Michael D. George, Vatinee Y. Bunya, Nora Sandorfi, Mina Massaro‐Giordano, Frederick B. Vivino

**Affiliations:** ^1^ Penn Sjögren's Center Philadelphia Pennsylvania; ^2^ Perelman School of Medicine, University of Pennsylvania Philadelphia; ^3^ Penn Dry Eye & Ocular Surface Center, Scheie Eye Institute, Perelman School of Medicine, University of Pennsylvania Philadelphia; ^4^ Department of Ophthalmology New York University Grossman School of Medicine New York; ^5^ Penn‐Presbyterian Medical Center, Perelman School of Medicine, University of Pennsylvania Philadelphia

## Abstract

**Objective:**

The goal was to assess the diagnostic performance of three novel autoantibodies (NA) for Sjögren disease (SjD) by comparing NA prevalence in patients with SjD, other autoimmune rheumatic diseases (ARDs), nonspecific chronic sialadenitis (CS), and controls.

**Methods:**

We identified rheumatology outpatients with confirmed SjD, systemic lupus erythematosus (SLE), rheumatoid arthritis (RA), systemic sclerosis (SSc), CS, and healthy controls. Participants underwent serum testing for antibodies to salivary gland protein 1 (SP‐1), parotid secretory protein, and carbonic anhydrase‐6 (CA‐6). Participants with SjD, CS, and controls also underwent testing of saliva. Receiver operating characteristic (ROC) curve C‐statistics along with sensitivity, specificity, positive, and negative likelihood ratios were calculated for each serum autoantibody for SjD vs CS or controls.

**Results:**

Among the 468 patients screened, 444 were analyzed, including cohorts with SjD (n = 149), SLE (n = 70), SSc (n = 56), RA (n = 73), CS (n = 31), and control patients (n = 65). There was no statistical difference (*P* = 0.80) in the presence of one or more NA in the serum of patients with SjD compared with participants with other ARDs, CS, or control patients. ROC curves demonstrated poor discriminative ability for SjD versus CS or control patients for any positive NA (0.47) or any individual NA (range 0.44–0.53). The positive likelihood ratio for having any positive novel autoantibody was 1.04 and 0.83 for SjD vs CS or control patients, respectively.

**Conclusion:**

Testing serum for NA demonstrated a poor ability to distinguish patients with SjD from control patients or those with CS or other ARDs. This serum autoantibody panel is not likely to be useful as a diagnostic test for SjD.

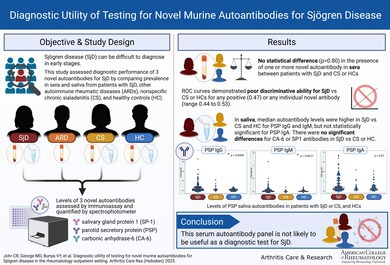

## Graphical abstract

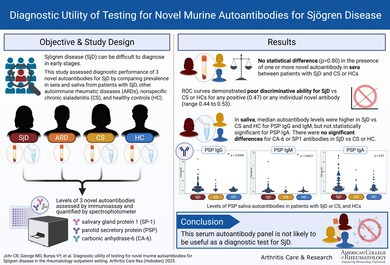

## INTRODUCTION

Sjögren disease (SjD) can be extremely difficult to diagnose in its early stages because of the heterogeneity of clinical presentations, need for extensive testing, and input required from multiple specialists as part of the evaluation.^1^

**SIGNIFICANCE & INNOVATIONS**

- This is the first study in the adult rheumatology literature to report the diagnostic performance of novel autoantibodies (NA) to salivary gland protein 1, parotid secretory protein, and carbonic anhydrase‐6 in a well‐characterized cohort of patients with Sjögren disease (SjD) fulfilling accepted classification criteria.
- This is one of the first studies to test for the presence of NA in the sera of several well‐defined cohorts of non‐SjD autoimmune rheumatic diseases, including systemic lupus erythematosus, rheumatoid arthritis, and systemic sclerosis.
- This is the first study to compare the prevalence of the NA in the saliva of patients with SjD vs healthy control patients and those with chronic sialadenitis.
- This study is particularly relevant for everyday clinical practice because it includes two separate well‐defined control groups, including one with sicca symptoms and nonspecific inflammation on minor salivary gland biopsy.

Recent classification criteria for SjD^2^, ^3^, ^4^ were designed to define homogeneous populations of patients for clinical trials and other studies but were not intended to facilitate diagnosis. Nevertheless, classification criteria are frequently used by clinicians to diagnose SjD by default because of the lack of universally accepted diagnostic criteria. Patients with seronegative SjD (negative anti‐SSA) comprise approximately one‐third of the overall group and are at the highest risk of misclassification because minor salivary gland biopsies are often not offered to or are refused by patients. This diagnostic challenge underscores the need to identify novel biomarkers that may be present when established biomarkers are not. As with other autoimmune rheumatic diseases (ARDs), therapeutic measures for SjD are most likely to be successful when initiated in the early stages before permanent irreversible damage occurs to the exocrine glands and other organs.

Previous work has convincingly demonstrated that the novel murine autoantibodies antiparotid secretory protein (PSP), antisalivary protein 1 (SP‐1), and anticarbonic anhydrase 6 (CA‐6) are markers of early disease in the interleukin‐14α transgenic mouse model for SjD because these autoantibodies appear in the blood earlier in the disease course than anti‐SSA/SSB.^5^ These novel autoantibodies (NA) have also been detected in the nonobese diabetic mouse model for SjD and in humans with established SjD.^5^, ^6^ It was also observed that among 20 patients with SjD who had positive biopsies but lacked anti SSA or SSB, the NA anti–SP‐1 and anti‐CA6 were present in 45% and 5% of cases, respectively,^5^ which suggests a possible role for diagnosis in seronegative cases.

Despite limited evidence, a diagnostic panel to test serum for the NA or “Early Sjögren's Profile,” also known as “Sjö,” became commercially available in 2013 (Immco Diagnostics, Inc., Buffalo, NY) and has been used by some specialists in clinical practice to diagnose “early SjD.” Several studies and case reports in the eye literature provided evidence that the “Early Sjögren's Profile” can be used to screen patients with dry eye for SjD, and, when positive, may provide a rationale for early referral to other specialists for further diagnostic evaluation.^7^, ^8^, ^9^, ^10^, ^11^, ^12^, ^13^

The purpose of the present study was to investigate the prevalence of NA in patients with known SjD, related autoimmune diseases, SjD mimics (eg, chronic nonspecific sialadenitis), and nonautoimmune controls to determine the sensitivity, specificity, likelihood ratios, and discriminative ability of the “Early Sjögren's Profile” as a diagnostic test.

## METHODS

### Patient selection and inclusion/exclusion criteria

The electronic medical record was used to screen for *International Statistical Classification of Diseases, Tenth Revision* (ICD‐10) codes among established patients at two large rheumatology outpatient practices within the same university health system. Participants with ICD‐10 codes from rheumatology visits corresponding to SjD, systemic lupus erythematosus (SLE), rheumatoid arthritis (RA), systemic sclerosis (SSc) and dry mouth/nonspecific chronic sialadenitis (CS) were identified. All records were reviewed by rheumatologists to confirm diagnoses, and patients were observed for at least one year. Control participants included established patients with osteoarthritis, fibromyalgia, osteoporosis, other non‐ARDs, or healthy volunteers. Patients with incomplete or unconfirmed diagnoses and control participants with a history of dry eyes and/or dry mouth, other autoimmune diseases, family history of autoimmune diseases, or previous referral for antinuclear antibody positivity were excluded from the study. Control participants and those with CS with positive anti‐SSA antibodies on study assays were excluded from analysis.

### Sjögren evaluation

Patients with suspected SjD routinely underwent a complete history and physical examination, serological testing, measurement of unstimulated whole‐mouth salivary flow, salivary scintigraphy,^14^, ^15^ an unanesthetized Schirmer test, and vital dye staining of the ocular surface. Patients who tested negative for anti‐SSA were asked to undergo a labial minor salivary gland biopsy. All patients with a diagnosis of SjD met one or more classification criteria for SjD,^2^, ^3^, ^4^ demonstrated objective evidence of dry eyes and dry mouth and either anti‐SSA positivity and/or a positive labial minor salivary gland biopsy (focal lymphocytic sialadenitis with a focus score ≥1/4 mm^2^). Information on which SjD criteria were met was not available, but to assess impact of isolated anti‐SSB antibodies, a sensitivity analysis was performed, excluding five patients with positive anti‐SSB but negative anti‐SSA antibodies. Patients diagnosed with nonspecific CS^16^ demonstrated objective evidence of dry eyes and/or dry mouth and had abnormal biopsies but failed to meet histologic and classification criteria for SjD.

### Study design and specimen collection

After signing informed consent and Health Insurance Portability and Accountability Act (HIPPA) authorization, all participants completed a medical questionnaire and specimen collection during one study visit. Recorded information included history of dry eyes and dry mouth, medical diagnoses, demographics, medications, associated autoimmune diseases, and family history of autoimmune or connective tissue disease. Additional information was collected on patients with known SjD regarding the duration of xerostomia, dry eye, and extraglandular or internal organ manifestations. Serum samples were obtained from all participants, and saliva samples were obtained from patients with SjD, CS, and control participants.

### Specimen processing

Blood specimens for all eligible participants were collected in one serum separator red top tube and centrifuged for storage at −80°C until shipment. Additionally, for some participants, random whole‐mouth salivary flow rates (sialometry) were measured (15‐minute collection) after swallowing once at the time of blood specimen collection. Saliva specimen collection tubes were weighed on an analytical balance pre‐ and post‐collection to determine the volume of saliva produced (1 g saliva = 1 mL). A protease inhibitor cocktail was added in proportion to the saliva sample volume (10 μL of 100 × protease inhibitor cocktail per 1 mL saliva). Specimen collection tubes were then vortexed for 5 to 10 seconds to homogenize the samples for storage at −80°C until shipment.

### Novel autoantibody assay

The tests were performed as a solid phase immunoassay by Immco Diagnostics Reference Laboratory (Trinity Biotech, Inc.) in Buffalo, New York. All specimens were anonymously coded and laboratory personnel were blinded to patient diagnoses. Briefly, microtiter plates were coated with recombinant antigen (CA‐6, PSP, or salivary gland protein 1 [SP1]) proteins expressed and purified from *Escherichia coli*, followed by blocking the unreacted sites to reduce nonspecific binding. Control participant and patient serum samples were incubated in the antigen coated wells that allow specific antibodies present in the serum to bind to the antigen. Unbound antibody and other serum proteins were removed by washing the microtiter plate. Bound antibodies were detected by adding horseradish peroxidase labeled anti‐human IgA, IgG, or IgM conjugate to the plates. Unbound conjugate is removed by washing. A specific enzyme substrate 3,3′,5,5′‐tetramethylbenzidine (TMB) was then added to the wells, and the presence of antibodies was detected by a color change produced by the conversion of the TMB substrate to a colored reaction product. The reaction was stopped and the intensity of the color change, which is proportional to the concentration of antibody, was read by a spectrophotometer at 450 nm. Results were expressed in enzyme‐linked immunosorbent assay (ELISA) units per milliliter (EU/mL) and reported as positive or negative, with positive defined as ≥20 EU/mL. Salivary autoantibodies were evaluated using the same methods and expressed as EU/mL without a specific cut‐off defined for positivity.

### Statistical analysis

Characteristics of patients with SjD, SLE, SSc, RA, CS, and control patients were compared descriptively. Differences in the frequency of novel serum autoantibody positivity across these diagnoses was assessed by Fisher exact testing (given small cell frequencies). Bonferroni correction was applied for multiple comparisons, with an adjusted *P* value of 0.0056 (0.05 divided by 9) considered statistically significant across all analyses given the nine antibodies studied. Receiver operating characteristic (ROC) curve C‐statistics were used to evaluate the ability of each serum autoantibody (dichotomized as positive or negative) to discriminate patients with SjD versus either CS or control participants, first with these two groups combined to maximize power, and then versus each individually. Comparison versus CS was thought to mimic how testing might be used in clinical practice, whereas comparisons versus control participants included a population with less likelihood of misclassification (ie, patients with CS who may actually have or develop SjD). Sensitivity, specificity, and positive and negative likelihood ratios (LR) were calculated for each serum autoantibody for SjD versus control participants or SjD versus CS. C‐statistics were then recalculated using each autoantibody as a continuous measure to assess whether a different cut‐off might lead to different performance. In a sensitivity analysis, novel serum autoantibody positivity was also assessed for each group of patients among those who were SSA negative to assess the utility of these autoantibodies for SjD diagnosis among patients who were SSA negative (a specific group for whom NA may be particularly useful). An additional sensitivity analysis excluded those with CS and control participants with rheumatoid factor (RF) ≥20 or antinuclear antibody (ANA) ≥1:80 to address any potential misclassification.

For novel salivary autoantibodies, dot plots were used to visualize the optical density for patients with SjD, CS, or control participants given the lack of an established cut‐off for positivity. Medians were compared using Kruskal‐Wallis testing given the skewed nature of the data (SjD vs CS or control participants given lower sample size). Again, ROC C‐statistics were used to assess the ability of salivary autoantibody values to distinguish SjD vs CS/control participants, with continuous values used for autoantibodies. In this analysis, the largest differences between groups were found for PSP salivary antibodies. As an exploratory analysis, a binary cut‐off for each of PSP IgG, IgM, and IgA was chosen as the 95th percentile value for each antibody among control participants, not including patients with CS (in whom autoantibody levels tended to be slightly higher). We calculated C‐statistics, sensitivity, specificity, and positive and negative LR for distinguishing SjD versus CS or control participants using these binary definitions of salivary PSP antibodies. These analyses were repeated among patients with a negative SSA antibody.

All analyses were performed using Stata version 15.1 (College Station, TX: StataCorp LLC). The study was approved by the University of Pennsylvania Institutional Review Board.

## RESULTS

A total of 468 patients with diagnoses of interest were recruited. After excluding patients who did not meet the classification criteria, including 3 patients with possible SjD, 1 patient with biopsy‐proven CS who also met Sjögren classification criteria, 3 control participants or patients with CS with anti‐SSA antibodies, and 17 patients who did not complete antibody testing, there were 444 patients for analysis, including 149 with SjD, 70 with SLE, 56 with SSc, 73 with RA, 31 with CS, and 65 control participants (non‐ARD or healthy volunteers). All groups were primarily female, and patients with SLE and control participants were younger than the other patient groups (Table 1). SSA antibody positivity was most common among those with SjD (61.7%).

**Table 1:** Cohort characteristics*

Characteristic	Primary SjD	Lupus	SSc	RA	CS	Control
N	149	70	56	73	31	65
Female, n (%)	143 (96.0)	66 (94.3)	53 (94.6)	57 (78.1)	29 (93.5)	52 (80.0)
Age, mean (SD), y	60.8 (13.7)	48.7 (15.3)	58.7 (12.5)	59.2 (12.8)	60.8 (16.6)	41.8 (20.5)
Dry eye, n (%)	143 (96.0)	42 (60.0)	37 (66.1)	27 (37.0)	26 (83.9)	0 (0.0)
Dry mouth, n (%)	144 (96.6)	38 (54.3)	37 (66.1)	29 (39.7)	26 (83.9)	0 (0.0)
ANA ≥ 1:40, n (%)	104 (69.8)	64 (91.4)	53 (94.6)	55 (75.3)	13 (41.9)	23 (35.4)
ANA ≥ 1:80, n (%)	96 (64.4)	64 (91.4)	52 (92.9)	47 (64.4)	11 (35.5)	15 (23.1)
RF IgM ≥ 10, n (%)	66 (44.3)	30 (42.9)	27 (48.2)	60 (82.2)	9 (29.0)	21 (32.3)
RF IgM ≥ 20, n (%)	45 (30.2)	19 (27.1)	15 (26.8)	51 (69.9)	6 (19.4)	8 (12.3)
Anti‐SSA, n (%)	92 (61.7)	25 (35.7)	18 (32.1)	7 (9.6)	0 (0.0)	0 (0.0)
Anti‐SSB, n (%)	48 (32.2)	9 (12.9)	4 (7.1)	6 (8.2)	2 (6.5)	2 (3.1)

* ANA, antinuclear antibody; CS, chronic sialadenitis; RA, rheumatoid arthritis; RF, rheumatoid factor; SjD, Sjögren disease; SSc, systemic sclerosis.

### Novel serum autoantibodies

The frequency of each novel serum autoantibody by disease is shown in Table 2. At least one serum autoantibody was positive in 43.6% of those with SjD, 44.3% with SLE, 51.8% with SSc, 45.2% with RA, 41.9% with CS, and 52.3% of control participants, with no significant difference across diseases (*P* = 0.80). Among individual serum autoantibodies, significant differences in CA‐6 IgG were seen (*P* < 0.001, statistically significant after Bonferroni correction), but this was driven by higher rates among those with SLE, SSc, and RA. There were no significant differences between SjD and CS or between SjD and control participants across any of the antibodies (all *P* > 0.05). Results were similar in a sensitivity analysis limited to patients with SjD with a negative anti‐SSA (Supplemental Table 1), as well in analyses excluding five patients with SjD with anti‐SSB but negative anti‐SSA, and in analyses excluding CS and control participants with RF ≥20 or ANA ≥1:80 (not shown). Among those with SLE, SSc, or RA, frequencies of NA were also similar in those with and without dry eye (*P* = 0.83) and those with and without dry mouth (*P* = 0.34). No significant differences in the prevalence of NA were observed among patients with SjD classified by salivary flow rate (ie, <0.2 cc/min or >0.2 cc/min).

**Table 2:** Novel serum autoantibody positivity by disease*

Variable	Primary Sjögren disease	Lupus	SSc	RA	CS	Control	Primary SjD vs CS or control participants ROC (95% CI)
N	149	70	56	73	31	65	
CA‐6 IgG, n (%)^a^	11 (7.4)	16 (22.9)	19 (33.9)	18 (24.7)	5 (16.1)	7 (10.8)	0.47 (0.44–0.51)
CA‐6 IgM, n (%)	21 (14.1)	7 (10.0)	6 (10.7)	4 (5.5)	3 (9.7)	13 (20.0)	0.49 (0.44–0.53)
CA‐6 IgA, n (%)	5 (3.4)	3 (4.3)	2 (3.6)	2 (2.7)	3 (9.7)	4 (6.2)	0.48 (0.45–051)
CA‐6 any, n (%)	33 (22.1)	22 (31.4)	23 (41.1)	22 (30.1)	10 (32.3)	22 (33.8)	0.44 (0.39–0.50)
PSP IgG, n (%)	5 (3.4)	5 (7.1)	4 (7.1)	3 (4.1)	3 (9.7)	(0.0)	0.50 (0.48–0.52)
PSP IgM, n (%)	10 (6.7)	1 (1.4)	1 (1.8)	(0.0)	1 (3.2)	8 (12.3)	0.49 (0.45–0.52)
PSP IgA, n (%)	16 (10.7)	4 (5.7)	4 (7.1)	5 (6.8)	2 (6.5)	2 (3.1)	0.53 (0.50–0.56)
PSP any, n (%)	29 (19.5)	9 (12.9)	7 (12.5)	8 (11.0)	5 (16.1)	10 (15.4)	0.52 (0.47–0.57)
SP‐1 IgG, n (%)	6 (4.0)	4 (5.7)	1 (1.8)	4 (5.5)	4 (12.9)	3 (4.6)	0.48 (0.45–0.51)
SP‐1 IgM, n (%)	16 (10.7)	9 (12.9)	10 (17.9)	6 (8.2)	(0.0)	14 (21.5)	0.48 (0.44–0.52)
SP‐1 IgA, n (%)	11 (7.4)	1 (1.4)	2 (3.6)	2 (2.7)	(0.0)	3 (4.6)	0.52 (0.49–0.55)
SP‐1 any, n (%)	31 (20.8)	13 (18.6)	12 (21.4)	12 (16.4)	4 (12.9)	19 (29.2)	0.48 (0.43–0.54)
Any of the above, n (%)	65 (43.6)	31 (44.3)	29 (51.8)	33 (45.2)	13 (41.9)	34 (52.3)	0.47 (0.41–0.54)

* Positive serum autoantibody defined as ≥20. ROC is the receiver operating characteristic curve C‐statistic for distinguishing patients with primary SjD from patients with CS or control participants. All *P* > 0.05 for pairwise comparison between SjD and CS and between SjD and controls. CA‐6, anticarbonic anhydrase 6; CI, confidence interval; CS, chronic sialadenitis; PSP, antiparotid secretory protein; RA, rheumatoid arthritis; SjD, Sjögren disease; SP‐1, antisalivary protein 1; SSc, systemic sclerosis.

^a^ Fisher's exact *P* < 0.001 for CA‐6 IgG, *P* = 0.006 for PSP IgM, and *P* = 0.021 for SP‐1 IgM when examining differences across all diagnosis groups, with only CA‐6 IgG meeting statistical significance after Bonferroni correction (*P* < 0.0056).

ROC curve C‐statistics for the ability of having any novel serum antibody to discriminate patients with SjD from those with CS or control participants was 0.47 (95% confidence interval [CI] 0.41–0.54) (worse than chance, with a greater frequency of the antibody in CS/control participants) and for individual autoantibodies ranged from 0.44 to 0.53, demonstrating minimal discriminative ability (Table 2). Results were similar when comparing SjD versus CS (Supplemental Table 2) or SjD versus control participants (Supplemental Table 3), with poor discrimination indicated by C‐statistics. For detection of any positive NA, the positive LR was 1.04 (95% CI 0.66–1.64) and 0.83 (95% CI 0.62–1.12) for SjD versus CS or control participants, respectively, and negative LR was 0.97 (95% CI 0.70–1.35) and 1.18 (0.88–1.58). For individual autoantibodies, all negative LR were >0.5, and all positive LR were <2 except for rare cases in which autoantibody frequency was low and sensitivity poor (Supplemental Tables 2 and 3).

### Novel salivary autoantibodies

**Figure 1:**
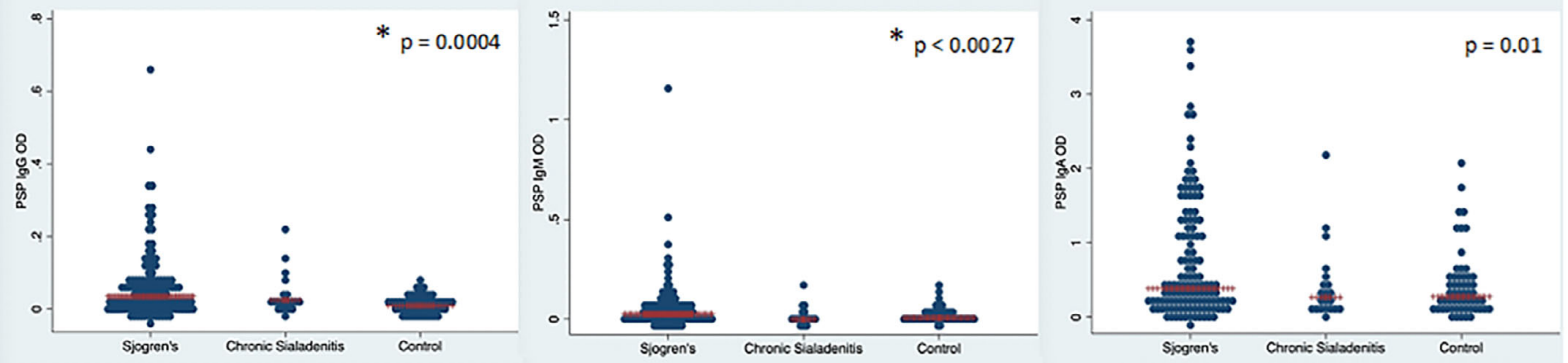
Levels of PSP saliva autoantibodies in patients with SjD, CS, or control participants. Dotplots show optical densities of novel saliva autoantibodies with red hashes representing medians for each group. *Kruskal‐Wallis testing comparing values in patients with SjD with those with CS or control participants showed higher levels in patients with SjD for PSP IgG and PSP IgM (both *P* <0.0056, statistically significant after Bonferroni correction). PSP IgA *P* = 0.01 not statistically significant after Bonferronni correction. CS, chronic sialadenitis; PSP, parotid secretory protein; SjD, Sjögren disease.

Salivary autoantibodies were available in 127 patients with SjD, 21 with CS, and 57 control participants. Dot plots showing levels of these autoantibodies in each group are in Figure 1. Median level autoantibody levels were higher in SjD versus CS and control participants for PSP IgG (*P* = 0.0004) and PSP IgM (*P* = 0.0027), whereas differences in PSP IgA did not meet statistical significance after Bonferroni correction (*P* = 0.01). There were no significant differences for CA6 or SP1 antibodies in SjD vs CS and control participants. ROC curve C‐statistics were also highest for PSP salivary antibodies and were 0.65 (95% CI 0.57–0.72), 0.63 (95% CI 0.55–0.70), and 0.60 (95% CI 0.53–0.68) for PSP IgG, IgM, and IgA salivary autoantibodies, respectively (Supplemental Table 4). Using the 95th percentile in controls (not including patients with CS) as a cut‐point for positive/negative, PSP salivary autoantibodies were more common in SjD than in CS or control participants (Table 3); sensitivity for SjD was 39.4%, 16.5%, and 18.9% with positive LR of 5.12, 4.30, and 4.91 for IgG, IgM, and IgA autoantibodies, respectively. Positivity of any PSP salivary autoantibody had sensitivity of 44.9% and positive LR of 3.50. Results were similar among patients with SjD with a negative anti‐SSA (Supplemental Table 5).

**Table 3:** Performance of PSP saliva autoantibodies using a cut‐point at levels above the 95th percentile in control participants*

Parameter	Primary SjD, N = 127	CS, N = 21	Control, N = 57	Test characteristic for SjD vs CS or control participants				
				Sens (95% CI)	Spec (95% CI)	LR+ (95% CI)	LR− (95% CI)	ROC (95% CI)
PSP IgG saliva >0.0537, n (%)^a^	50 (39.4)	4 (19.0)	2 (3.5)	39.4% (30.8–48.4)	92.3% (84.0–97.1)	5.12 (2.30–11.40)	0.66 (0.56–0.77)	0.66 (0.61–0.71)
PSP IgM saliva > 0.0994, n (%)	21 (16.5)	1 (4.8)	2 (3.5)	16.5% (10.5–24.2)	96.2% (89.2–99.2)	4.30 (1.33–13.90)	0.87 (0.79–0.95)	0.56 (0.52–0.60)
PSP IgA saliva > 1.4819, n (%)	24 (18.9)	1 (4.8)	2 (3.5)	18.9% (12.5–26.8)	96.2% (89.2–99.2)	4.91 (1.54–15.80)	0.84 (0.77–0.93)	0.58 (0.54–0.62)
Any PSP saliva >95th percentile, n (%)^a^	57 (44.9)	5 (23.8)	5 (8.8)	44.9% (36.1–54.0)	87.2% (77.7–93.7)	3.50 (1.90–6.44)	0.63 (0.53–0.76)	0.66 (0.60–0.72)

* The ROC C‐statistic is based on a binary cut‐off for defining a positive test. CI, confidence interval; LR+, positive likelihood ratio; LR−, negative likelihood ratio; PSP, antiparotid specific protein; ROC, receiver operating characteristic curve C‐statistic; Sens, sensitivity; SjD, Sjögren disease; Spec, specificity.

^a^ Fisher's exact comparing across all three groups *P* < 0.001 for PSP IgG, *P* = 0.02 for PSP IgM, *P* = 0.007 for PSP IgA, and <0.001 for any PSP saliva antibody >95th percentile in healthy control participants. Differences for PSP, IgG, and any PSP antibody meet statistical significance after Bonferroni correction (*P* < 0.0056, still accounting for nine comparisons given that nine total salivary antibodies were measured).

## DISCUSSION

Novel biomarkers for SjD that appear early in the disease course or that can be detected in patients who lack anti‐SSA are sorely needed. In this study we evaluated the diagnostic utility of three NA for SjD: anti–SP‐1, anti–CA‐6, and anti‐PSP. We found that the NA have no significant diagnostic value to distinguish SjD from healthy control participants or those with CS in the rheumatology practice setting, with LRs near 1 and a receiver operating characteristic curve C‐statistic very close to 0.5. In addition, the NA were not useful in distinguishing between SjD and other systemic ARDs, including RA, SLE, and SSc. The prevalence of NA in patients with other ARDs was not significantly different in patients with and without sicca symptoms. Thus, this panel would be unlikely to accurately predict the presence of secondary or associated SjD among these latter individuals.

The literature regarding the utility of the NA up to this point has been inconsistent. Some studies suggest evidence of potential diagnostic utility with only SP‐1,^6^, ^8^, ^17^ other studies with only CA‐6,^18^ and yet others with only PSP.^19^ One Chinese study suggests potential diagnostic utility with all three NA.^20^ The only study outside of ours that reports on the diagnostic performance of the NA was performed on children and demonstrates that the NA were not helpful in distinguishing between patients with SjD and control participants.^21^ The potential reasons for the inconsistent results in the literature are myriad. First of all, early studies included both mice and humans and were initially measured by Western blot.^5^, ^6^, ^17^ As the testing for NA became commercialized and more widespread, the assay was switched from Western blot to ELISA, and it is conceivable that this change in assays resulted in a loss of specificity. Next, most NA publications involve only a small number of participants. Many do not specify whether those with SjD met any classification criteria. Control groups, if present, were often not well‐defined. It is notable that we found similar rates of NA positivity in patients with SjD compared to previous studies, but importantly, we also found high rates of NA positivity in control participants.^5^ Last, differences in geography may also help explain some of the inconsistencies that have been reported.

The NA are also marketed as early markers of SjD. It seems this is based largely on evidence from mouse models. Previous authors have noted that to evaluate this suggestion more thoroughly, longitudinal studies are needed. However, the presence of autoantibodies is typically maintained in systemic autoimmune diseases. For example, in one longitudinal registry study from Sweden, serum autoantibodies were detected in SjD up to 18 years before diagnosis, and the prevalence of RF, ANA and anti‐SSA/SSB actually increased over time.^22^ Because of this, we would expect to find significantly higher levels of NA in those with “late” or established SjD compared with controls, but this was not the case in our study. Additionally, we found no differences in NA prevalence among patients with SjD classified as “early” or “advanced” SjD based on salivary flow rates.

It has also been suggested that the NA may be diagnostically helpful for cases in which established seromarkers (ie, anti‐SSA/SSB) for SjD are negative and labial salivary biopsy declined. With this in mind, a sensitivity analysis was performed for the confirmed participants with SjD who had a negative SSA but a positive labial salivary biopsy (n = 58). The results were essentially unchanged.

Because autoantibody formation in SjD occurs locally in exocrine glands,^23^ we also tested saliva for the NA. We observed statistically higher levels of PSP in saliva samples from patients with SjD compared with those with CS or control participants. However, the relative insensitivity of this finding renders this observation less useful as the basis for a future diagnostic test. Furthermore, the specimen processing for saliva required at the present time is more labor intensive than a simple blood test and further diminishes its potential diagnostic value.

Our study has multiple strengths. First, this is one of the largest NA studies to date with well‐defined cohorts. All participants with SjD met modern classification criteria, and patients with other connective tissue disorders were diagnosed by rheumatologists at an academic center. The “normal” control group bore little relation to SjD. By definition, they had no sicca symptoms and no personal nor family history of autoimmune disease. These were largely patients with osteoarthritis, osteoporosis, chronic pain syndrome (with negative previous evaluations) or healthy volunteers. The CS control group included patients who presented with sicca symptoms, underwent a thorough evaluation for SjD, and exhibited evidence of nonspecific inflammation on labial salivary biopsy but who were SSA negative and did not meet classification criteria for SjD. This “practical” control group represented patients for whom the NA test would be typically applied. In addition, we tested for the prevalence of NA in autoimmune diseases related to SjD to see if the NA could help distinguish between SjD and other closely related connective tissue disorders. Similarly, it could not. Last, all participants were observed for at least a year to ensure that they remained as initially classified.

Our study has limitations. This was not a longitudinal study and does not conclusively prove that levels of NA do not diminish in SjD over time. Additionally, this is a single center study, and evaluations with more than one center have demonstrated positive results. It was a single‐specialty study involving only rheumatology outpatients. As such, it may not be applicable in other contexts. We used one or more among three different sets of classification criteria (2016 American College of Rheumatology [ACR]/EULAR, 2012 ACR, and 2002 American‐European Consensus Group) to classify the participants as SjD and were unable stratify results by which criteria was met.^2^, ^3^, ^4^ However, many individuals fulfilled more than one set of classification criteria, and all participants with SjD were either anti‐SSA positive or had a positive labial minor salivary gland biopsy with objective evidence of dry eyes and dry mouth. Previous studies have documented only small differences in sensitivity and specificity when different criteria sets are applied to the same population with SjD.^24^ We also found similar results after excluding five patients who were anti‐SSB positive but anti‐SSA negative. These observations, therefore, make it unlikely that exclusive use of the 2016 ACR/EULAR classification criteria^4^ would have significantly altered our results. It is possible that some patients with CS could have had SjD with negative biopsy, but we also included a separate control group and conducted sensitivity analyses excluding those with CS or controls with low titer ANA and RF with similar results. Future studies should be longitudinal, re‐evaluating patients years later to see if there has been any change in the NA levels or the underlying diagnoses. Investigators could look at whether NA are associated with a particular subset of patients with SjD such as those with interstitial lung disease or renal tubular acidosis. Measuring NA in saliva has potential diagnostic utility and could be further explored with additional testing in more cohorts with SjD, healthy normal controls, and other groups.

In summary, based on the analysis of NA data in this large, well‐defined cohort, we conclude that testing for the NA with the present commercial assay is not useful in distinguishing between SjD and non‐SjD in the outpatient rheumatology setting. The NA are not likely to be helpful for diagnosing SjD in practice nor useful for inclusion in further classification criteria of SjD for clinical research.

## AUTHOR CONTRIBUTIONS

All authors contributed to at least one of the following manuscript preparation roles: conceptualization AND/OR methodology, software, investigation, formal analysis, data curation, visualization, and validation AND drafting or reviewing/editing the final draft. As corresponding author, Dr Johr confirms that all authors have provided the final approval of the version to be published and takes responsibility for the affirmations regarding article submission (eg, not under consideration by another journal), the integrity of the data presented, and the statements regarding compliance with institutional review board/Declaration of Helsinki requirements.

## Supporting information


**Disclosure Form**:

**Supplemental Table 1** Novel Antibody Positivity in Patients with a Negative SSA Antibody
**Supplemental Table 2:** Test Characteristics for Novel Serum Autoantibodies for Distinguishing Patients with Primary Sjögren's Disease from Chronic Sialadenitis
**Supplemental Table 3:** Test Characteristics for Novel Serum Autoantibodies for Distinguishing Patients with Primary Sjögren's Disease from Controls
**Supplemental Table 4:** Salivary Antibody Values and ROC in Patients with Primary Sjögren's vs. Chronic Sialadenitis or Controls
**Supplemental Table 5:** Performance of PSP Saliva Antibodies at Levels Above the 95^th^ Percentile in Controls Among Patients with a Negative SSA

## References

[1] Vivino FB . Diagnosis and evaluation of Sjögren's syndrome. In: Vivino FB , ed. Sjögren's Syndrome: A Clinical Handbook. Elsevier; 2019:21–35.

[2] Vitali C , Bombardieri S , Jonsson R , et al; European Study Group on Classification Criteria for Sjögren's Syndrome. Classification criteria for Sjögren's syndrome: a revised version of the European criteria proposed by the American‐European Consensus Group. Ann Rheum Dis 2002;61(6):554–558. doi:10.1136/ard.61.6.554 12006334 PMC1754137

[3] Shiboski SC , Shiboski CH , Criswell L , et al; Sjögren's International Collaborative Clinical Alliance (SICCA) Research Groups. American College of Rheumatology classification criteria for Sjögren's syndrome: a data‐driven, expert consensus approach in the Sjögren's International Collaborative Clinical Alliance cohort. Arthritis Care Res (Hoboken) 2012;64(4):475–487. doi:10.1002/acr.21591 22563590 PMC3349440

[4] Shiboski CH , Shiboski SC , Seror R , et al; International Sjögren's Syndrome Criteria Working Group. 2016 American College of Rheumatology/European League Against Rheumatism Classification Criteria for Primary Sjögren's Syndrome: A Consensus and Data‐Driven Methodology Involving Three International Patient Cohorts. Arthritis Rheumatol 2017;69(1):35–45. doi:10.1002/art.39859 27785888 PMC5650478

[5] Shen L , Suresh L , Lindemann M , et al. Novel autoantibodies in Sjogren's syndrome. Clin Immunol 2012;145(3):251–255. doi:10.1016/j.clim.2012.09.013 23123440

[6] Shen L , Kapsogeorgou EK , Yu M , et al. Evaluation of salivary gland protein 1 antibodies in patients with primary and secondary Sjogren's syndrome. Clin Immunol 2014;155(1):42–46. doi:10.1016/j.clim.2014.08.009 25178982

[7] Bunya VY , Massaro‐Giordano M , Vivino FB , et al. Prevalence of novel candidate Sjögren syndrome autoantibodies in the Penn Sjögren's International Collaborative Clinical Alliance Cohort. Cornea 2019;38(12):1500–1505. doi:10.1097/ICO.0000000000002147 31517725 PMC6832820

[8] Bunya VY , Ying GS , Maguire MG , et al; DREAM Study Research Group. Prevalence of novel candidate Sjogren syndrome autoantibodies in the Dry Eye Assessment and Management (DREAM) study. Cornea 2018;37(11):1425–1430. doi:10.1097/ICO.0000000000001714 30161055 PMC6173620

[9] Matossian C , Micucci J . Characterization of the serological biomarkers associated with Sjögren's syndrome in patients with recalcitrant dry eye disease. Clin Ophthalmol 2016;10:1329–1334. doi:10.2147/OPTH.S106973 27499612 PMC4959586

[10] Beckman KA . Detection of early markers for Sjögren syndrome in dry eye patients. Cornea 2014;33(12):1262–1264. doi:10.1097/ICO.0000000000000278 25343702

[11] Vishwanath S , Everett S , Shen L , et al. Xerophthalmia of Sjogren's syndrome diagnosed with anti‐salivary gland protein 1 antibodies. Case Rep Ophthalmol 2014;5(2):186–189. doi:10.1159/000364941 25076899 PMC4105945

[12] Vishwanath S , Shen L , Suresh L , et al. Anti‐salivary gland protein 1 antibodies in two patients with Sjogren's syndrome: two case reports. J Med Case Rep 2014;8(1):145. doi:10.1186/1752-1947-8-145 24885364 PMC4031912

[13] Everett S , Vishwanath S , Cavero V , et al. Analysis of novel Sjogren's syndrome autoantibodies in patients with dry eyes. BMC Ophthalmol 2017;17(1):20. doi:10.1186/s12886-017-0412-8 28270126 PMC5341407

[14] Hermann GA , Vivino FB , Shnier D , et al. Diagnostic accuracy of salivary scintigraphic indices in xerostomic populations. Clin Nucl Med 1999;24(3):167–172. doi:10.1097/00003072-199903000-00006 10069726

[15] Hermann GA , Vivino FB , Goin JE . Scintigraphic features of chronic sialadenitis and Sjögren's syndrome: a comparison. Nucl Med Commun 1999;20(12):1123–1132. doi:10.1097/00006231-199912000-00004 10664993

[16] Kassimos DG , Shirlaw PJ , Choy EH , et al. Chronic sialadenitis in patients with nodal osteoarthritis. Br J Rheumatol 1997;36(12):1312–1317. doi:10.1093/rheumatology/36.12.1312 9448593

[17] Xuan J , Wang Y , Xiong Y , et al. Investigation of autoantibodies to SP‐1 in Chinese patients with primary Sjögren's syndrome. Clin Immunol 2018;188:58–63. doi:10.1016/j.clim.2017.12.008 29292085

[18] Karakus S , Baer AN , Agrawal D , et al. Utility of novel autoantibodies in the diagnosis of Sjögren's syndrome among patients with dry eye. Cornea 2018;37(4):405–411. doi:10.1097/ICO.0000000000001471 29504954

[19] Karakus S , Baer AN , Akpek EK . Clinical correlations of novel autoantibodies in patients with dry eye. J Immunol Res 2019;2019:7935451. doi:10.1155/2019/7935451 30766890 PMC6350592

[20] Jin Y , Li J , Chen J , et al. Tissue‐specific autoantibodies improve diagnosis of primary Sjögren's syndrome in the early stage and indicate localized salivary injury. J Immunol Res 2019;2019:3642937. doi:10.1155/2019/3642937 31205955 PMC6530237

[21] Thatayatikom A , Jun I , Bhattacharyya I , et al. The diagnostic performance of early Sjögren's syndrome autoantibodies in juvenile Sjögren's syndrome: The University of Florida Pediatric Cohort Study. Front Immunol 2021;12:704193. doi:10.3389/fimmu.2021.704193 34249010 PMC8267463

[22] Theander E , Jonsson R , Sjöström B , et al. Prediction of Sjögren's syndrome years before diagnosis and identification of patients with early onset and severe disease course by autoantibody profiling. Arthritis Rheumatol 2015;67(9):2427–2436. doi:10.1002/art.39214 26109563

[23] Du W , Han M , Zhu X , et al. The multiple roles of B cells in the pathogenesis of Sjögren's syndrome. Front Immunol 2021;12:684999. doi:10.3389/fimmu.2021.684999 34168653 PMC8217880

[24] Le Goff M , Cornec D , Jousse‐Joulin S , et al. Comparison of 2002 AECG and 2016 ACR/EULAR classification criteria and added value of salivary gland ultrasonography in a patient cohort with suspected primary Sjögren's syndrome. Arthritis Res Ther 2017;19(1):269. doi:10.1186/s13075-017-1475-x 29208023 PMC5717850

