# Supplementary material for: Diagnostic Use of Testing for Novel Murine Autoantibodies for Sjögren Disease in the Rheumatology Outpatient Setting
Source: Arthritis Care Res (Hoboken). 2026 Mar 2;78(7):875–82. doi: 10.1002/acr.70005 (PMC13313094; doi:10.1002/acr.70005)
Supplement: Supplementary file 2 — Supplemental Table 1 Novel Antibody Positivity in Patients with a Negative SSA Antibody Supplemental Table 2: Test Characteristics for Novel Serum Autoantibodies for Distinguishing Patients with Primary Sjögren's Disease from Chronic Sialadenitis Supplemental Table 3: Test Characteristics for Novel Serum Autoantibodies for Distinguishing Patients with Primary Sjögren's Disease from Controls Supplemental Table 4: Salivary Antibody Values and ROC in Patients with Primary Sjögren's vs. Chronic Sialadenitis or Controls Supplemental Table 5: Performance of PSP Saliva Antibodies at Levels Above the 95th Percentile in Controls Among Patients with a Negative SSA [file ACR-78-875-s002.docx]

**Supplemental Table 1:** Novel Antibody Positivity in Patients with a Negative SSA Antibody

|  | Primary Sjögren's  (SSA neg) | Lupus  (SSA neg) | Systemic Sclerosis  (SSA neg) | Rheumatoid Arthritis  (SSA neg) | Chronic Sialadenitis | Control |
| --- | --- | --- | --- | --- | --- | --- |
| N | 57 | 45 | 38 | 66 | 31 | 65 |
| CA-6 IgG* | 4 (7.0%) | 7 (15.6%) | 15 (39.5%) | 16 (24.2%) | 5 (16.1%) | 7 (10.8%) |
| CA-6 IgM | 6 (10.5%) | 5 (11.1%) | 4 (10.5%) | 4 (6.1%) | 3 (9.7%) | 13 (20.0%) |
| CA-6 IgA | 2 (3.5%) | 2 (4.4%) | 0 (0.0%) | 1 (1.5%) | 3 (9.7%) | 4 (6.2%) |
| CA-6 Any | 12 (21.1%) | 12 (26.7%) | 17 (44.7%) | 20 (30.3%) | 10 (32.3%) | 22 (33.8%) |
| PSP IgG | 2 (3.5%) | 3 (6.7%) | 1 (2.6%) | 3 (4.5%) | 3 (9.7%) | 0 (0.0%) |
| PSP IgM | 5 (8.8%) | 1 (2.2%) | 1 (2.6%) | 0 (0.0%) | 1 (3.2%) | 8 (12.3%) |
| PSP IgA | 5 (8.8%) | 3 (6.7%) | 4 (10.5%) | 4 (6.1%) | 2 (6.5%) | 2 (3.1%) |
| PSP Any | 12 (21.1%) | 6 (13.3%) | 4 (10.5%) | 7 (10.6%) | 5 (16.1%) | 10 (15.4%) |
| SP-1 IgG | 2 (3.5%) | 3 (6.7%) | 1 (2.6%) | 3 (4.5%) | 4 (12.9%) | 3 (4.6%) |
| SP-1 IgM | 4 (7.0%) | 5 (11.1%) | 4 (10.5%) | 6 (9.1%) | 0 (0.0%) | 14 (21.5%) |
| SP-1 IgA | 5 (8.8%) | 0 (0.0%) | 2 (5.3%) | 2 (3.0%) | 0 (0.0%) | 3 (4.6%) |
| SP-1 Any | 11 (19.3%) | 8 (17.8%) | 6 (15.8%) | 11 (16.7%) | 4 (12.9%) | 19 (29.2%) |
| Any of the above | 24 (42.1%) | 17 (37.8%) | 20 (52.6%) | 29 (43.9%) | 13 (41.9%) | 34 (52.3%) |

Positive defined as ≥20

* Fisher’s exact test p = 0.001 for CA-6 IgG, p = 0.02 for PSP IgM, and p = 0.03 for SP-1 IgM when examining differences across all diagnosis groups, with only CA-6 IgG meeting statistical significance after Bonferroni correction (p < 0.0056).

All p > 0.05 for pairwise comparison between Primary Sjögren's and chronic sialadenitis and all p > 0.05 between Primary Sjögren's and controls except for SP-1 IgM (p = 0.04, not meeting statistical significance after Bonferroni correction)

PSP = anti-parotid specific protein, SP-1 = anti-salivary protein 1, CA-6 = anti-carbonic anhydrase 6

**Supplemental Table 2:** Test Characteristics for Novel Serum Autoantibodies for Distinguishing Patients with Primary Sjögren's Disease from Chronic Sialadenitis

|  | Primary Sjögren's | Chronic Sialadenitis | Sensitivity  (95% CI) | Specificity  (95% CI) | LR+  (95% CI) | LR-  (95% CI) | ROC binary  (95% CI) | ROC*  (95% CI) |
| --- | --- | --- | --- | --- | --- | --- | --- | --- |
| N | 149 | 31 |  |  |  |  |  |  |
| CA-6 IgG | 11 (7.4%) | 5 (16.1%) | 7.4%  (4.2-13.6) | 83.9% (66.3-94.5) | 0.46  (0.17-1.22) | 1.10  (0.94-1.30) | 0.46  (0.39-0.53) | 0.28  (0.19-0.36) |
| CA-6 IgM | 21 (14.1%) | 3 (9.7%) | 14.1%  (8.9-20.6) | 90.3% (74.2-98.0) | 1.46  (0.46-4.58) | 0.95  (0.83-1.09) | 0.52  (0.46-0.58) | 0.49  (0.39-0.59) |
| CA-6 IgA | 5 (3.4%) | 3 (9.7%) | 3.4%  (1.1-7.6) | 90.3% (74.2-98.0) | 0.35  (0.09-1.38) | 1.07  (0.95-1.21) | 0.47  (0.41-0.52) | 0.38  (0.28-0.49) |
| CA-6 Any | 33 (22.1%) | 10 (32.3%) | 22.1% (16.2-30.2) | 67.7% (48.6-83.3) | 0.69  (0.38-1.24) | 1.15  (0.90-1.49) | 0.45  (0.36-0.54) | - |
| PSP IgG | 5 (3.4%) | 3 (9.7%) | 3.4%  (1.1-7.6) | 90.3% (74.2-98.0) | 0.35  (0.09-1.38) | 1.07  (0.95-1.21) | 0.47  (0.41-0.52) | 0.46  (0.35-0.57) |
| PSP IgM | 10 (6.7%) | 1 (3.2%) | 6.7%  (3.2-11.9) | 96.8% (83.3-99.9) | 2.08  (0.28-15.70) | 0.96  (0.89-1.04) | 0.52  (0.48-0.55) | 0.50  (0.39-0.60) |
| PSP IgA | 16 (10.7%) | 2 (6.5%) | 10.7%  (6.2-16.7) | 93.6% (78.6-99.2) | 1.66  (0.40-6.87) | 0.95  (0.86-1.06) | 0.52  (0.47-0.57) | 0.53  (0.42-0.64) |
| PSP Any | 29 (19.5%) | 5 (16.1%) | 19.5% (13.3-26.6) | 83.9% (66.3-94.5) | 1.21  (0.51-2.87) | 0.96  (0.81-1.14) | 0.52  (0.44-0.59) | - |
| SP-1 IgG | 6 (4.0%) | 4 (12.9%) | 4.0%  (1.5-8.5) | 87.1% (70.2-96.4) | 0.31  (0.94-1.04) | 1.10  (0.96-1.27) | 0.56  (0.40-0.52) | 0.46  (0.34-0.58) |
| SP-1 IgM | 16 (10.7%) | 0 (0.0%) | 10.7%  (6.2-16.7) | 100.0% (N/A) | - | 0.89  (0.84-0.94) | 0.55  (0.53-0.58) | 0.63  (0.52-0.73) |
| SP-1 IgA | 11 (7.4%) | 0 (0.0%) | 7.4%  (3.7-12.7) | 100.0% (N/A) | - | 0.93  (0.88-0.97) | 0.54  (0.52-0.56) | 0.54  (0.45-0.64) |
| SP-1 Any | 31 (20.8%) | 4 (12.9%) | 22.8% (14.5-28.0) | 87.1% (70.2-96.4) | 1.61  (0.61-4.24) | 0.91  (0.78-1.07) | 0.54  (0.47-0.61) | - |
| Any of the above | 65 (43.6%) | 13 (41.9%) | 43.6% (35.9-52.3) | 58.1% (39.1-75.5) | 1.04  (0.66-1.64) | 0.97  (0.70-1.35) | 0.51  (0.41-0.61) | - |

* ROC (Receiver operating characteristic curve C-statistic) if antibody values are considered continuous rather than as a binary cut-off at 20, only calculated for individual antibody results.

LR+ = positive likelihood ratio, LR- = negative likelihood ratio, PSP = anti-parotid specific protein, SP-1 = anti-salivary protein 1, CA-6 = anti-carbonic anhydrase 6

**Supplemental Table 3:** Test Characteristics for Novel Serum Autoantibodies for Distinguishing Patients with Primary Sjögren's Disease from Controls

|  | Primary Sjögren's | Control | Sensitivity  (95% CI) | Specificity  (95% CI) | LR+  (95% CI) | LR-  (95% CI) | ROC binary  (95% CI) | ROC*  (95% CI) |
| --- | --- | --- | --- | --- | --- | --- | --- | --- |
| N | 149 | 65 |  |  |  |  |  |  |
| CA-6 IgG | 11 (7.4%) | 7 (10.8%) | 7.4%  (4.2-13.6) | 89.2%  (79.1-95.6) | 0.69  (0.28-1.69) | 1.04  (0.94-1.14) | 0.48  (0.44-0.53) | 0.39  (0.31-0.48) |
| CA-6 IgM | 21 (14.1%) | 13 (20.0%) | 14.1%  (8.9-20.6) | 80.0%  (68.2-88.9) | 0.70  (0.38-1.32) | 1.07  (0.94-1.23) | 0.47  (0.41-0.53) | 0.41  (0.33-0.49) |
| CA-6 IgA | 5 (3.4%) | 4 (6.2%) | 3.4%  (1.1-7.6) | 93.8%  (85.0-98.3) | 0.54  (0.15-1.97) | 1.03  (0.96-1.10) | 0.49  (0.45-0.52) | 0.45  (0.37-0.54) |
| CA-6 Any | 33 (22.1%) | 22 (33.8%) | 22.1%  (16.2-30.2) | 66.2%  (53.4-77.4) | 0.65  (0.42-1.03) | 1.18  (0.97-1.43) | 0.44  (0.37-0.51) | - |
| PSP IgG | 5 (3.4%) | 0 (0.0%) | 3.4%  (1.1-7.6) | 100.0%  (N/A) | - | 0.97  (0.94-1.00) | 0.52  (0.50-0.53) | 0.53  (0.45-0.61) |
| PSP IgM | 10 (6.7%) | 8 (12.3%) | 6.7%  (3.2-11.9) | 87.7%  (77.2-94.5) | 0.54  (0.23-1.32) | 1.06  (0.96-1.18) | 0.47  (0.43-0.52) | 0.37  (0.29-0.45) |
| PSP IgA | 16 (10.7%) | 2 (3.1%) | 10.7%  (6.2-16.7) | 96.9%  (89.3-99.6) | 3.49  (0.83-14.70) | 0.92  (0.86-0.99) | 0.54  (0.51-0.57) | 0.54  (0.46-0.63) |
| PSP Any | 29 (19.5%) | 10 (15.4%) | 19.5% (13.3-26.6) | 84.6%  (73.5-92.4) | 1.27  (0.66-2.44) | 0.95  (0.84-1.08) | 0.52  (0.47-0.57) | - |
| SP-1 IgG | 6 (4.0%) | 3 (4.6%) | 4.0%  (1.5-8.5) | 95.4%  (87.1-99.0) | 0.87  (0.22-3.38) | 1.01  (0.94-1.07) | 0.50  (0.47-0.53) | 0.42  (0.34-0.50) |
| SP-1 IgM | 16 (10.7%) | 14 (21.5%) | 10.7%  (6.2-16.7) | 78.5%  (66.5-87.7) | 0.50  (0.26-0.96) | 1.14  (0.99-1.31) | 0.45  (0.39-0.50) | 0.37  (0.29-0.45) |
| SP-1 IgA | 11 (7.4%) | 3 (4.6%) | 7.4%  (3.7-12.7) | 95.4%  (87.1-99.0) | 1.60  (0.46-5.54) | 0.97  (0.90-1.04) | 0.51  (0.48-0.55) | 0.53  (0.45-0.61) |
| SP-1 Any | 31 (20.8%) | 19 (29.2%) | 22.8%  (14.5-28.0) | 70.8%  (58.2-81.4) | 0.71  (0.44-1.16) | 1.12  (0.94-1.34) | 0.46  (0.39-0.52) | - |
| Any of the above | 65 (43.6%) | 34 (52.3%) | 43.6%  (35.9-52.3) | 44.9%  (35.1-60.5) | 0.83  (0.62-1.12) | 1.18  (0.88-1.58) | 0.46  (0.38-0.53) | - |

* ROC (Receiver operating characteristic curve C-statistic) if antibody values are considered continuous rather than as a binary cut-off at 20, only calculated for individual antibody results.

LR+ = positive likelihood ratio, LR- = negative likelihood ratio, PSP = anti-parotid specific protein, SP-1 = anti-salivary protein 1, CA-6 = anti-carbonic anhydrase 6

**Supplemental Table 4:** Salivary Antibody Values and ROC in Patients with Primary Sjögren's vs. Chronic Sialadenitis or Controls

|  | Primary Sjögren's | Controls or chronic sialadenitis | ROC*  (95% CI) |
| --- | --- | --- | --- |
| N | 127 | 78 |  |
| CA-6 IgG saliva | 0.015  [0.00, 0.045] | 0.014  [0.004, 0.036] | 0.50  (0.42-0.57) |
| CA-6 IgM saliva | 0.032  [0.011, 0.068] | 0.028  [0.004, 0.061] | 0.54  (0.46-0.63) |
| CA-6 IgA saliva | 0.435  [0.212, 0.842] | 0.391  [0.235, 0.708] | 0.52  (0.44-0.60) |
| PSP IgG saliva | 0.035  [0.005, 0.081] | 0.016  [-0.002, 0.028] | 0.65  (0.57-0.72) |
| PSP IgM saliva | 0.025  [-0.005, 0.069] | 0.002  [-0.009, 0.038] | 0.63  (0.55-0.70) |
| PSP IgA saliva | 0.387  [0.172, 1.277] | 0.272  [0.128, 0.541] | 0.60  (0.53-0.68) |
| SP-1 IgG saliva | 0.014  [0.004, 0.040] | 0.011  [0.001, 0.024] | 0.57  (0.49-0.65) |
| SP-1 IgM saliva | 0.047  [0.012, 0.085] | 0.051  [0.021, 0.089] | 0.47  (0.39-0.55) |
| SP-1 IgA saliva | 0.368  [0.156, 0.883] | 0.308  [0.149, 0.538] | 0.58  (0.50-0.66) |

Median [Interquartile range] for optical densities shown for each antibody

*ROC = receiver operating characteristic curve C-statistic

PSP: Anti-parotid specific protein, SP-1: anti-salivary protein 1, CA-6: anti-carbonic anhydrase 6

**Supplemental Table 5:** Performance of PSP Saliva Antibodies at Levels Above the 95^th^ Percentile in Controls Among Patients with a Negative SSA

|  |  |  | Test characteristic for Sjögren's vs. Chronic sialadenitis or Controls | | | | |
| --- | --- | --- | --- | --- | --- | --- | --- |
|  | Primary Sjögren's  (SSA neg)  N = 50 | Chronic Sialadenitis or Control  N = 78 | Sens | Spec | LR+ | LR- | ROC vs. either (binary) |
| PSP IgG saliva > 0.0537* | 19 (38.0%) | 6  (7.7%) | 38.0%  (24.7-52.8) | 92.3%  (84.0-97.1) | 4.94  (2.12-11.5) | 0.67  (0.54-0.84) | 0.65  (0.58-0.73) |
| PSP IgM saliva > 0.0994 | 9  (18.0%) | 3  (3.8%) | 18.0%  (8.6-31.4) | 96.2%  (89.2-99.2) | 4.68  (1.33-16.50) | 0.85  (0.74-0.98) | 0.57  (0.51-0.63) |
| PSP IgA saliva > 1.4819 | 7  (14.0%) | 3  (3.8%) | 14.0%  (5.8-26.7) | 96.2%  (89.2-99.2) | 3.64  (0.99-13.4) | 0.89  (0.79-1.01) | 0.55  (0.50-0.60) |
| Any PSP saliva >95^th^ percentile* | 22 (44.0%) | 10  (12.8%) | 44.0%  (30.0-58.7) | 87.2%  (77.7-93.7) | 3.43  (1.78-6.63) | 0.64  (0.50-0.83) | 0.66  (0.58-0.74) |

*Fischer’s exact test comparing across all 3 groups p < 0.001 for PSP IgG, p = 0.032 for PSP IgM, p = 0.136 for PSP IgA, and < 0.001 for any PSP saliva antibody > 95^th^ percentile in healthy controls. Differences for PSP IgG and any PSP antibody meet statistical significance after Bonferroni correction (p < 0.0056, still accounting for 9 comparisons given that 9 total salivary antibodies were measured).

Sens = sensitivity, Spec = specificity, LR+ = positive likelihood ratio, LR- = negative likelihood ratio, ROC = receiver operating characteristic curve C-statistic, PSP = anti-parotid specific protein
